# Editorial: Cellular senescence in physiology and pathophysiology

**DOI:** 10.3389/fphys.2023.1173284

**Published:** 2023-03-22

**Authors:** Mustafa Ark, Mohammad Nasir Uddin

**Affiliations:** ^1^ Department of Pharmacology, Faculty of Pharmacy, Gazi University, Ankara, Turkey; ^2^ Department of Medical Physiology, Texas A&M Health Science Center School of Medicine/Baylor Scott & White Hospital, Temple, TX, United States

**Keywords:** cellular senescence, SASP, senolytic, senomorphic, senotherapeutic

It has been known since Leonard Hayflick’s observations and brilliant designed experiments that cells have a limited replicative capacity and lifespan (Hayflick and Moorhead, 1961). Cells that reach a certain number of doubling times can no longer divide and subsequently these cells turn into a special cellular state called senescent (Özdemir et al., 2023). The heterogeneity of senescent cells has limited the knowledge of drivers and consequences of cellular senescence in tissues and organs (Roy et al., 2020). This cellular response, which is characterized by an irreversible cell cycle arrest after limited cell divisions is called replicative senescence. Sir Macfarlane Burnett called this phenomenon “*the Hayflick limit*” (Burnet, 1974). Subsequent studies have also shown that senescence is induced by various intrinsic and extrinsic stress factors such as oxidative stress, UV radiation, oncogenes and anticancer compounds or radiation used cancer therapy other than this replicative form (Özdemir et al., 2023). Although senescent cells are metabolically active, they differ morphologically and functionally from normal cells. Senescent cells are larger than normal cells and show multiple and large nuclei. These cells secrete a large number of biologically active molecules, mainly cytokines, growth factors, and matrix metalloproteinases, called SASP (Senescence associated secretory phenotype) into their microenvironment. SASP has been shown to cause deleterious effects such as inflammation, cancer cell proliferation, migration, invasion, and resistance to chemotherapeutics in the surrounding tissues (Coppé et al., 2010; Özdemir et al., 2023). All these features of senescent cells have been associated with many physiopathological events, especially age-related diseases and cancer. However, despite all these studies, there are many contradictory demonstrations that need to be clarified regarding the development of senescence, the morphological features of senescent cells, and the effects of SASP on other cells around.

While it is generally accepted that senescent cells have an enlarged and flattened morphological structure, the 3D holographic measurements indicate that there was no difference in the thickness between senescent and normal cells (Şimay et al., 2018).

An interesting study published in JCB revealed that senescent cancerous cells phagocytose both neighboring senescent and non-senescent tumor cells in their microenvironment (Tonnessen-Murray et al., 2019). However, this feature of senescent cancer cells could not be confirmed by other studies (Şimay Demir et al., 2021).

Functionally, the effects of SASP on other surrounding cells vary depending on the cell types and senescence stimuli. It also differs on which type of cells it interacts with, and whether these cells are normal or cancerous (Basisty et al., 2020). Therefore, a comprehensive profiling of SASP is required in each senescence form to identify the effects of SASP and to take action against the detrimental consequences it triggers (Basisty et al., 2020; Jochems et al., 2021).

The transcriptome signatures of the senescent cell shows heterogeneity depending on the cell type and the stress stimulant (Hernandez-Segura et al., 2017). After senescence induced in a panel of 13 cancer cell lines, transcriptome analysis of senescent cells was performed and these data were presented in an interactive online system called “CANCER SENESCopedia” (Jochems et al., 2021). These reports show that there is yet a long way to go in determining the characteristic features of senescent cells in general. Since these studies were performed in a range of cancer cell lines, information on senescence formation induced by different stimuli in both non-tumour cells and other cancer types is limited.

On the other hand, studies on the discovery and development of senotherapeutic drugs that target the specific elimination of senescent cells (senolytic) and suppression of the activity of SASP (senomorphic) to inhibit the deleterious results of senescence in both aging and cancer chemotherapy, continue.

This Research Topic of Frontiers in Physiology includes important studies on senescence and some of the various physiopathological events that play an important role in the process. The article by Zhang et al. points out that polo-like kinase 1 activity delays senescence formation of nucleus pulposus cells and mediates the reduction of intervertebral disc degeneration and thus the prevention of back pain (Zhang et al., 2022). Apart from this, senescence is also associated with parturition. Wan et al. explain the role of fetal lung-associated exosomes from the amniotic fluid in primary human amniotic epithelial cell senescence and apoptosis thereby inducing delivery (Wan et al.). Ren et al. contribute to this Research Topic by identifying a novel sulfur dioxide probe that inhibits high glucose-induced senescence in umbilical vascular endothelial cells by inducing the degradation of lipid droplets (Ren et al.). Senescence is also induced after adjuvant therapy (chemotherapy and radiotherapy) by inducing DNA damage. In their study, Jiang et al. demonstrated that the small GTPases RagC and Rheb are required for the activation of mTOR to promote chemoresistance in chemotherapy-induced senescence in HepG2 cells (Jiang et al.). Moreover, the regulatory role of E3 ubiquitin ligases is discussed in response to DNA damage, as reviewed by Lu et al..

In conclusion, recent reports of this Research Topic fill an important gap in the physiopathological events in which senescent takes place.
